# Structure-Functional Activity Relationship of β-Glucans From the Perspective of Immunomodulation: A Mini-Review

**DOI:** 10.3389/fimmu.2020.00658

**Published:** 2020-04-22

**Authors:** Biao Han, Kartik Baruah, Eric Cox, Daisy Vanrompay, Peter Bossier

**Affiliations:** ^1^Laboratory of Aquaculture & Artemia Reference Center, Faculty of Bioscience Engineering, Ghent University, Gent, Belgium; ^2^Department of Animal Nutrition and Management, Faculty of Veterinary Medicine and Animal Sciences, Swedish University of Agricultural Sciences, Uppsala, Sweden; ^3^Laboratory of Immunology, Faculty of Veterinary Medicine, Ghent University, Merelbeke, Belgium; ^4^Laboratory of Immunology and Animal Biotechnology, Faculty of Bioscience Engineering, Ghent University, Gent, Belgium

**Keywords:** β-glucans, structure-function relationship, immunomodulation, molecular structure, molecular weight, solubility

## Abstract

β-Glucans are a heterogeneous group of glucose polymers with a common structure comprising a main chain of β-(1,3) and/or β-(1,4)-glucopyranosyl units, along with side chains with various branches and lengths. β-Glucans initiate immune responses via immune cells, which become activated by the binding of the polymer to specific receptors. However, β-glucans from different sources also differ in their structure, conformation, physical properties, binding affinity to receptors, and thus biological functions. The mechanisms behind this are not fully understood. This mini-review provides a comprehensive and up-to-date commentary on the relationship between β-glucans' structure and function in relation to their use for immunomodulation.

## Introduction

β-Glucans are a group of naturally occurring polysaccharides which are widely distributed in bacteria, fungi, algae, and cereals, in which they are part of the cell wall structure and have many other biological activities (1). Structurally, β-glucans are long or short-chain polymers of β-(1,3) or β-(1,4) linked glucose subunits which may be branched, with the side chains branching from the six-position of the backbone (2, 3). For example, β-glucans of mushrooms have short β-(1,6)-linked branches whereas those of yeast have β-(1,6)-side branches with additional β-(1,3) regions (4). Supplementary Figure 1 summarizes different chemical structures of β-glucans (5). Furthermore, β-glucans could also form secondary structures and the possibility of various structural forms could lead to differences in the mechanisms behind the immunomodulating activities (6, 7). The literature on immune responses to glucans can be quite confusing as what is observed for one preparation of glucan is often inappropriately extrapolated to all glucans. When discussing the immune-modulator functions of glucans, here we mostly considered β-1,3-glucan purified from fungal cell walls.

**Figure 1:**
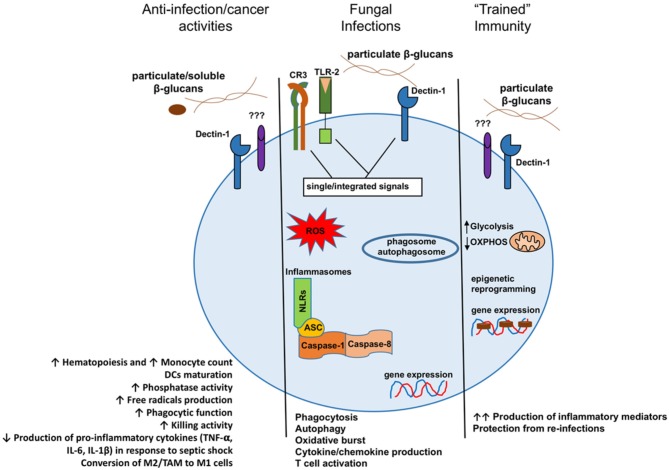
Model presenting the different consequences of β-glucan recognition by monomorphonuclear phagocytes in the context of antitumoral activities, fungal infection recognition, or the creation of innate immune memory. DCs, dendritic cells; M2, alternatively activated macrophages; TAM, tumor-associated macrophages; M1, classically activated macrophages. Reproduced from Camilli et al. (27) with permission and under the terms of the Creative Commons Attribution License (https://creativecommons.org/licenses/by/4.0/).

The immunomodulatory properties of β-glucans have long been recognized (8). The activation of the immune system through modulation by β-glucans is rather complex and depends on many factors that have not yet been fully revealed. β-Glucan is a key pathogen-associated molecular pattern (PAMP) that is detected upon fungal infection to trigger the host's immune responses in both vertebrates and invertebrates (9). The induction of cellular responses by β-glucans is a result of their specific interaction with several pattern recognition receptors (PRRs), such as Dectin-1, complement receptor 3 (CR3), selected scavenger receptors, and lactosylceramide (LacCer). Receptor binding triggers a signal transduction in monomorphonuclear phagocytes (e.g., macrophages, monocytes, dendritic cells, and natural killer cells) and neutrophils (10–12). The activity of these β-glucan receptors seems to be highly dependent on the cell types. Research demonstrated that neutrophil modulation by β-glucan is predominantly CR3 dependent while Dectin-1 is the most important β-glucan receptor on macrophages (13–15). Upon β-glucan binding to the lectin site of the CR3 on phagocytes and NK cells, the receptor was activated to enhance the cytotoxicity against iC3b-opsonized target cells, including tumors (16, 17). Recognition of β-glucan by Dectin-1 on macrophages activates the downstream signaling pathway. As a consequence of these signaling activations, Dectin-1 triggers phagocytosis, ROS generation, microbial killing, and cytokine production (18, 19). Moreover, recent studies demonstrated that pre-administration of β-glucans resulted in innate immune memory, protecting the mice against re-infection with a lethal *Escherichia coli* (20). Increased protection was related to the function of “trained” monocytes (21). Innate immune memory is defined as a heightened response to a secondary infection that can be exerted toward both homologous and heterologous microorganisms. For the underlying mechanisms, epigenetic modifications and metabolic reprogramming do play crucial roles (22–24). It is acknowledged that particulate β-glucans may be the optimal preparation to induce innate immune memory, whereas low molecular weight β-glucans (e.g., laminarin) do not favor a high response (25, 26). Figure 1 presents the different consequences of β-glucan recognition by monomorphonuclear phagocytes.

Previous reports indicate that immunomodulatory effects of glucans could be influenced by differences in their structural characteristics such as branching frequency, solubility, molecular weight, polymer charge, and conformation in solution (7, 28, 29). It is still unclear how structural differences of glucans might affect their biological functions. This is partly due to the use of β-glucan polymers with different structural characteristics. Research studies on β-glucan have continued to grow since the 1950s when studies on these biomolecules began to be published. According to the Scopus Database (see Supplementary Figure 2), as of 2019, 9,652 papers had been related to immunological activities of β-glucan while <1 fifth of the papers included the term “structure” in the title and/or abstract. Systematic studies providing structure-function relationships for immunostimulation by β-glucans are generally lacking. Different immunological effects have been described and this might be related to the use of untreated, denatured, and renatured β-(1,3)-D-glucans which all differ in their structure. Thus, incorrect conclusions can be easily drawn when comparing results obtained by using such diverse β-glucans. In Table 1, the relationship between β-glucan structure and observed immunomodulatory properties has been summarized. This mini-review will try to summarize up-to-date information on the structure-functional relationship of β-glucans in relation to immunomodulation.

**Table 1:** Relationship between β-glucan structure and observed immunomodulatory properties.

**Source**	**Structure**	**MW/DB/Conformation**	**Solubility**	**Animal/Cell type**	**Immunostimulatory Activity**	**References**
*Aureobasidium pullulans*	(1-3)-β-D-Glucan backbone with (1-6)-β-linked side chains	–	Water-soluble	Rats/ Peyer's patch (PP) cells	$\bar{↑}$ IL-5, IL-6, and IgA production, reduction in blood hemoglobin and hematocrit concentrations in rats	Tanioka et al. (30)
*Agaricus bisporus, Agaricus brasiliensis*	(1-6)-β-D-Glucan	2.9 × 10^4^ g/mol/ 4.5 × 10^4^ g/mol	Insoluble	Human THP-1 macrophages	$\bar{↑}$ Gene expression IL-1β, TNF-α, and proinflammatory control	Smiderle et al. (31)
*Antrodia camphorate*	(1-3)-β-D-dextran main chain having (1-6)-β-dextran side branches	10–10^3^ kDa/ Helical structure	Water-soluble	Human leukemic U937 cells/Sarcoma 180-bearing mice	$\bar{↓}$ proliferation of cancer cells; NK activity	Liu et al. (32)
*Pleurotus ostreatus*	(1-3)-β-D-glucan, heteroglucans	2200–2900 kDa/ 0.25	Soluble/ Insoluble	Lymphocyte	$\bar{↑}$ proliferation of lymphocyte	Synytsya et al. (33)
Chemically synthetized	Oligo-(1-3)-β-D-glucan-mannose	0.83-0.99 kDa/ Not helical structures	–	BALB/c mice	$\bar{↑}$ influx MO into the peritoneal cavity, phagocytic activity of peritoneal MO; $\bar{↓}$ % of lymphocytes, intra-peritoneal	Descroix et al. (34)
*Dictyophora indusiata*	(1-3)-β-D-Glucan backbone with (1-6)- β-linked side chains	480 kDa/ Triple-helix	Water-soluble	Kunming (KM) mice inoculated with S180 cells	$\bar{↑}$ Thymus and spleen indexes; $\bar{↑}$ serum IL-2, IL-6, and TNF-α	Deng et al. (35)
*Flammulina velutipes*	(1-3)-β-D-glucan	200 kDa/ Single helix		Sarcoma 180 tumor cell	$\bar{↑}$ expression of cytokines	Leung et al. (36)
*Sclerotium rolfsii*	(1-3)-β-D-Glucan substituted with single (1-6)-d-Glcp residues	1100 kDa/ 0.33/ Triple helix	–	Human monocytes	$\bar{↑}$ TNF-α in monocytes	Falch et al. (37)
*Schizophyllum commune*	(1-3)-β-D-glucan main chain with (1-6)-β-D-glucopyrano branch at every three repeating	10^2^-10^4^ kDa/ 0.33/Random coil conformation in dimethylsulfoxide	–	Human peripheral blood mononuclear cells	$\bar{↑}$ Expression of cytokines; $\bar{↑}$ NK cells' activity, etc	Yoneda et al. (38)
*Lentinus edodes*	(1-3)-β-D-Glucan backbone with (1-6)-β-linked side chains	1490 kDa/ Triple helix	Insoluble	BALB/c mice inoculated with S-180 cells	$\bar{↑}$ antitumor activity	Zhang et al. (39)
*Ganoderma lucidum*	(1-3)-β-D-Glucan (highly branched)	8 kDa	Water-soluble	CHO cells RAW264.7 cells; murine peritoneal MO;	$\bar{↑}$ MAPKs- and Syk-dependent TNF-α and IL-6; $\bar{↑}$ antitumor activity	Guo et al. (40)
*Poria cocos mycelia*	(1-3)-β-D-Glucan	26–268 kDa/ 0.39-0.96/ Single helix	Insoluble	Sarcoma 180 tumor cell	$\bar{↑}$ expression of cytokines	Lin et al. (41)
*Saccharomyces cerevisiae*	Liner-β-(1-3)-glucan	3.8 × 10^4^ g/mol	Water-insoluble, DMSO-soluble	Macrophage-like RAW264.7 cells	$\bar{↑}$ production of TNF-α and MCP-1	Zheng et al. (42)

*MW, molecular weight; DB, degree of branching; IL, interleukin; IgA, immunoglobulin A; TNF-α, tumor necrosis factor α; MO, macrophage; NK cell, natural killer cell; MCP-1, monocyte chemoattractant protein-1; $\bar{↑}$ increase, $\bar{↓}$ decrease*.

## Molecular Weight

Some evidences suggest that the immunomodulating activities of glucans are related to their molecular weight (MW), with higher MW glucans having more effect on the immune system. This is perhaps not so surprising as, in general, antigens with a higher MW are more immunogenic. However, maybe glucans with a high MW have a more stabilized structure and can be recognized directly by specific receptors on the surface of immune cells (43). Another influencing factor is the retention time in the intestinal system, where glucans are degraded and metabolized slowly. Differences in uptake from the intestinal lumen are dependent on their MW (44).

β-Glucans with a low MW and a short side chain (<5,000–10,000 MW) are commonly regarded as inactive (45). Besides, Brown and Gordon (44) demonstrated that immune cells can be directly activated by β-glucans with a high MW (such as zymosan), stimulating their phagocytic, cytotoxic, and antimicrobial activities, while low MW β-glucans need cytokines to modulate the immune response. The biological activities of a β-(1,3)-glucan isolated from *Grifola frandosa* changed with its MW, with the highest MW glucan always showing the most potent immunomodulatory effect (46). The same can also be applied to polysaccharide-K (Krestin, PSK,), a protein-bound polysaccharide obtained from basidiomycetes showing the strongest immunestimulating activities for PSK with the highest MW (>200 KDa) (47). However, controversial data on immunomodulating capacities of high molecular weight β-glucans vs. low molecular weight β-glucans still exist. For example, lentinan and schizophyllan, both pure β-(1,3)-glucans extracted from *Lentinus edodes* and *Schizophyllum commune*, respectively, exhibit the same antitumor activity against a murine cancer cell (Sarcoma 180) regardless of the use of a high or low MW form (48). Lei et al. (49), reported that a yeast β-glucan with low MW was better as an antioxidant and immunostimulant compared to the high MW form.

## Molecular Structure

### Backbone

*In vitro*, the murine Dectin-1 binding capacity of glucans in relation to their structural features was investigated by Adams et al. (50). It was demonstrated that the β-(1,3)-D-glucopyranosyl backbone of the glucans is essential for Dectin-1 recognition. Dectin-1 cannot recognize non-β-linked glucans (e.g., mannan or pullulan) and glucans isolated from plants (e.g., oats, barley, and wheat) who have a backbone of linear D-glucopyranosyl residues with a mixture of β-(1,3) and β-(1,4) linkages (51). Also, Dectin-1 did not interact with linear β-(1,3)-D-glucan oligosaccharides shorter than seven glucose subunits and β-1,3-glucans without any side chains (branches). Summarized, the minimal glucan subunit structure for Dectin-1 activation is a β-(1,3)-D-glucan oligosaccharide containing a backbone with at least seven glucose subunits and a single (1,6)-β-linked side-chain branch at the non-reducing end (50).

### Sidechain

The side chain length and branching frequency are also crucial for the immunomodulating ability of β-glucans (45). A previous study showed that glucans with only one single glucose molecule in the side chain had a lower macrophage activating ability than glucans extracted from the same yeast but with more glucose in the side chain (52). It has been reported that β-glucans with a branching ratio between 0.2 (1:5 branching)-−0.33 (1:3 branching) are most potent immunomodulators (9, 53). However, exceptions do exist as the binding affinity for CR3 between schizophyllan and scleroglucan differs greatly although they have a similar branching frequency. Also, when comparing the binding affinity of laminarin (1:10 branching) and schizophyllan (1:3 branching) for CR3, an insignificant difference was observed (21 μM vs. 11 μM) (1). Moreover, a branched β-glucan named pachyman, obtained from *Poria cocos*, has no anti-tumor activity, while debranched pachyman exhibits significant anti-tumor activity (54). A possible explanation might be that the glucans with a higher degree of branching could stereochemically interfere with each other, leading to less binding by specific receptors (15). The discrepancy of these results remains to be clarified.

### Conformation

Glucans can also form secondary structures, and this depends on the conformation of sugar residues, MW, and the inter- and intra-chain hydrogen-bonding (6). β-glucans exist in three conformations: single helix, triple helix and random coil. Whether a single helix or triple-helix β-(1-3)-D-glucan conformation has the highest biological activity is still an unresolved issue. The literature data appear inconsistent and are often contradictory. However, a single helix conformation is usually less stable than the triple helix conformation. *In vivo*, β-1,3-glucan assumes a triple-helical structure in which one β-1,3-glucan chain forms inter-strand hydrogen bonds with two other strands perpendicular to the axis of the triple helix. Thus, the triple helix conformation is the main structure in the cell wall of most fungi and recognition of the triple-helical β-1,3-glucan by an immune receptor is important for immune signaling (1, 55). Glucans with a single helix conformation showed a lower ability to suppress tumor growth (37) than glucans with a triple helix conformation. On the other hand, Saitô et al. (56), verified that β-(1–3)-D-glucans with a single strand chain showed a higher bioactivity than the β-glucans with a helix structure. The relationship between conformation and immunomodulatory properties of β-glucans suggest the existence of a biological system which can recognize the different conformations in the host's body. Hence, the relationship between glucan conformation and bioactivity still needs further study.

## Solubility

The physical properties of β-glucan, such as solubility, can also be impacted by molecular features, such as linkage pattern and molecular weight (57). Both soluble and particulate glucans have been reported to stimulate the immune response. For example, human whole blood incubated with soluble glucan isolated from yeast (Biotec Pharmacon ASA, Tromsø, Norway) showed an increased production of tumor necrosis factor alpha (TNF-α), Interleukin-6 (IL-6), the chemokine CXCL8 and the monocyte tissues factor (TF) (58). To date, soluble glucans, for their ease of delivery *in vivo*, have been widely used in clinical applications, whereas particulate glucans may be more effective in a local rather than systemic immunomodulatory effect (59). The difference can be explained by the use of different receptors by soluble and particulate β-glucans. Particulate β-glucans directly stimulate immune cell activation through Dectin-1 pathways while soluble glucans require a complement and CR3-dependent pathway activation for their antitumor activities (60). Moreover, Goodridge et al. (25) demonstrated that Dectin-1, in contrast to other PRRs, discriminates between soluble and particulate β-glucans. Phagocytosis and cytokine production by macrophages are only induced when Dectin-1 is bound to particulate β-glucan through the formation of a “phagocytic synapse” and the exclusion of regulatory phosphatases. This process represents a unique mechanism to discriminate PAMPs associated with a microbial surface.

## Particle Size

Particulate β-glucans can also be used as adjuvants for chemotherapy as well as adjuvants in vaccines for their additional effects on the immune system (61, 62). They could enhance hematopoietic responses in animal models under chemotherapy, by increasing interleukin-6 levels (63). Particulate β-glucans isolated from yeast are hollow, porous 2–4 μm spheres with an outer shell capable of mediating uptake by phagocytic cells. Therefore, the high payload of therapeutic agents, such as DNA, siRNA, protein/ peptide, and small molecules could be reduced by encapsulating these agents into the particles using a core-polyplex and layer-by-layer synthetic strategies and be applied to optimize the tumor microenvironment for cancer immunotherapy (64). For example, an *in situ* layer-by-layer syntheses of DNA-caged yeast β-glucan particles was shown to not only effectively protect the caged DNA from degradation but also facilitate the systemic delivery of the DNA content to macrophages *in vivo* (65, 66). The particle size of glucan matters and its generally known that nanoparticles with a diameter 1–2 μm are better absorbed by macrophages than large-size particles (67). However, a recent study showed that large (curdlan, up to 0.2 mm diameter) β-glucan-stimulated human dendritic cells (DCs) generate significantly more IL-1β, IL-6, and IL-23 compared to those stimulated with the smaller β-glucans (glucan microparticles; 1–5 μm diameter) (68). Additional studies are needed to investigate how the β-glucan size mediates the immune response.

## Conclusions and Perspectives

The structural and physical features of β-glucans determine their way of acting on the immune system. So, while describing the results of different experiments on the immunomodulatory properties of glucans, one should ideally provide a thorough description of the structural features of the glucans under study. Information on solubility, particle size, molecular weight, sidechain branching frequency and conformation should be provided. Also, we need well-characterized (1,3)-β-glucan polymers with varying structural characteristics when studying the influence of carbohydrates on the biological activity of glucans. Synthetic glucans could provide a unique opportunity to investigate the immunomodulating activities of glucans (50, 69). Also, a standardized commercial glucan with assured quality control (such as for instance a commercial glucan extracted from *Saccharomyces cerevisiae*) could be systematically used as a positive control, to compare the immunomodulatory activities with experimental glucans being tested. In addition, differences in reactivity of β-glucans in individuals and between different strains of test animals should also be taken into consideration when comparing studies (70).

It should also be noted that the isolation method may influence the characteristics of β-glucans and differences can be expected when glucans are isolated from the same sources (71). The most appropriate isolation method, dependent on the source of the β-glucan and the extraction procedure, must not affect the molecular integrity of the glucan and needs to guarantee product purity and optimal yield (72). Furthermore, chemical modification could be an effective way to enhance the biological activities of glucans. Several methods have been applied to change the physical properties of β-glucans (e.g., solubility) in order to improve their functional properties via chemical and physical cross-linking effects (such as carboxymethylation, sulfidation, and oxidation) (73, 74).

## Author Contributions

The review results from the discussion and the consensus of all authors listed BH, KB, EC, DV, and PB. The review was written by BH.

## Conflict of Interest

The authors declare that the research was conducted in the absence of any commercial or financial relationships that could be construed as a potential conflict of interest.
