# Supplementary material for: Structure-Functional Activity Relationship of β-Glucans From the Perspective of Immunomodulation: A Mini-Review
Source: Front Immunol. 2020 Apr 22;11:658. doi: 10.3389/fimmu.2020.00658 (PMC7188827; doi:10.3389/fimmu.2020.00658)
Supplement: Supplementary file 1 [file Data_Sheet_1.docx]

Supplementary Material

The Supplementary Material include two Supplementary Figures.


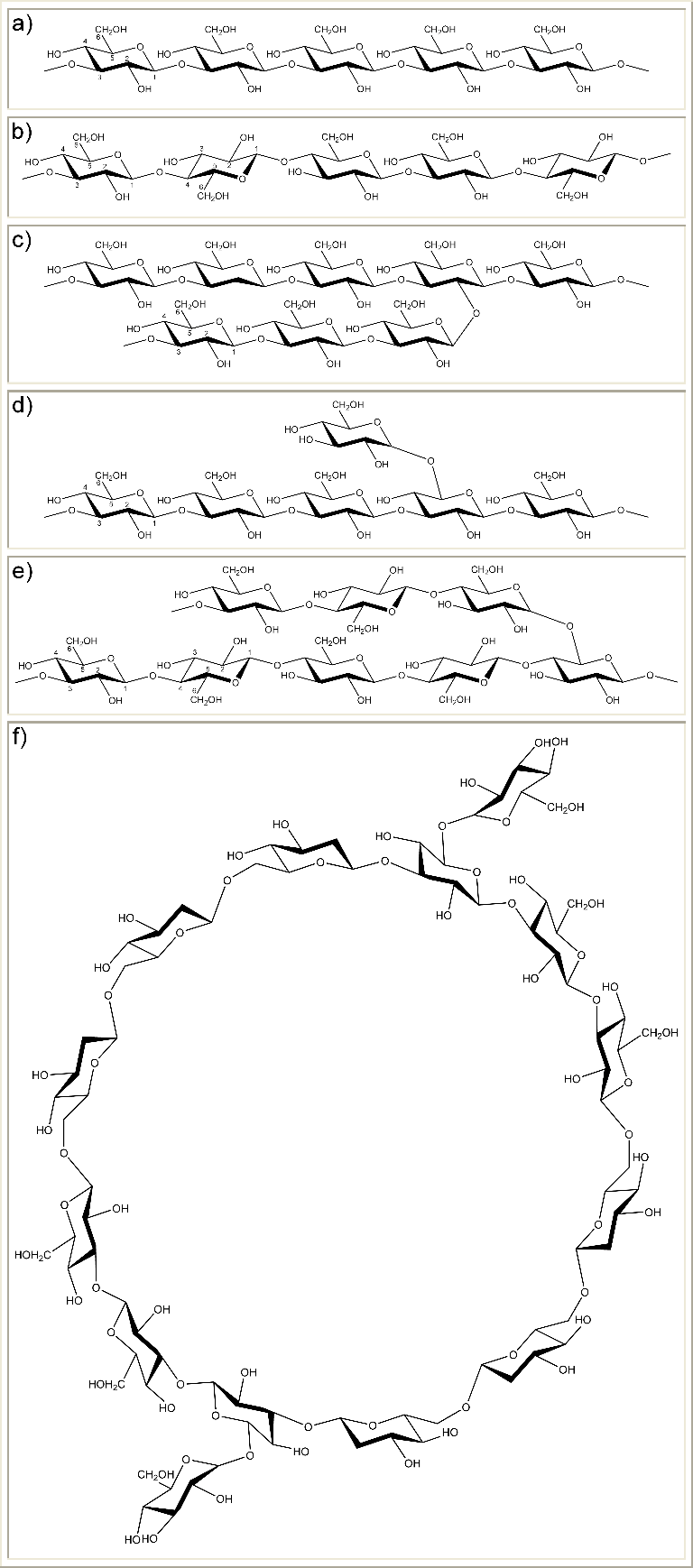


**Supplementary Figure 1.** Chemical structures of β-glucans. (a) Linear β-(1,3)-D-glucans; (b) Linear β-(1,3;1,4)-D-glucans; (c) Side-chain-branched β-(1,3;1,2)-D-glucans; (d) Side-chain-branched β-(1,3;1,6)-D-glucans; (e) Side-chain-branched β-(1,4;1,6)-D-glucans; (f) Cyclic β-(1,3;1,6)-glucans.

*Adapted from Barsanti et al. (2011) with permission from The Royal Society of Chemistry and Corresponding Author.*


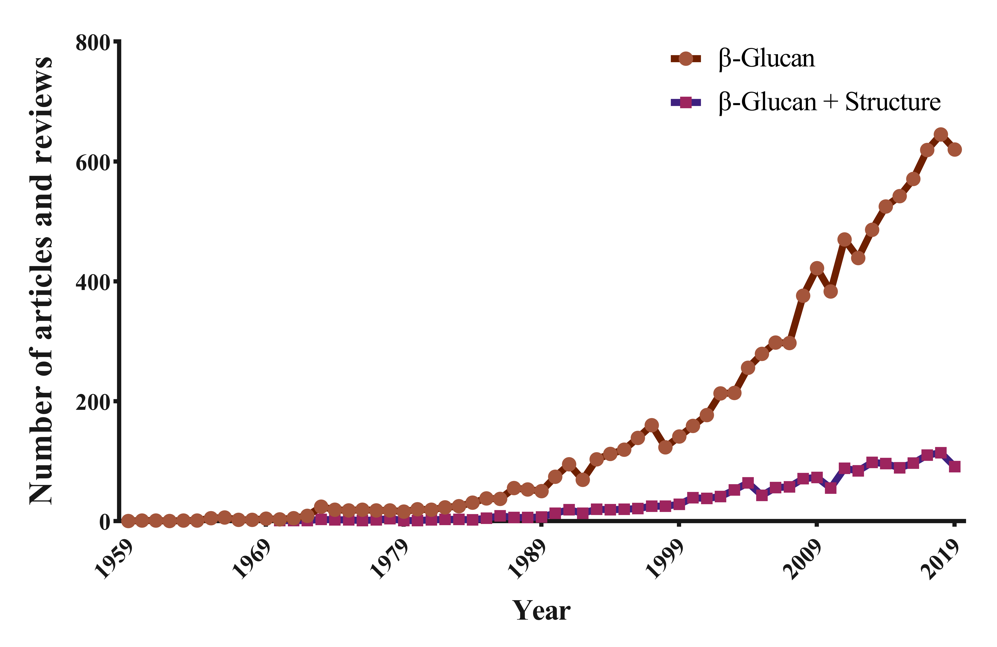
**Supplementary Figure 2.** Number of published articles and reviews in Scopus (<http://www.scopus.com>) from 1959-2019 using the term “β-Glucan” and “β-Glucan + Structure” in article titles, abstracts, and keywords.

**Reference**

Barsanti, L., Passarelli, V., Evangelista, V., Frassanito, A.M., and Gualtieri, P. (2011). Chemistry, physico-chemistry and applications linked to biological activities of beta-glucans. *Nat Prod Rep* 28**,** 457-466.
